# Innovative strategy for full-scale polar components explicition and ultrasonic-assisted optimization of *Astragalus membranaceus* flower

**DOI:** 10.1016/j.ultsonch.2025.107238

**Published:** 2025-01-24

**Authors:** Yumei Wang, Meiling Gu, Meng Zhang, Jialin Mao, Yujian Han, Qi Liu

**Affiliations:** a The Research Institute of Medicine and Pharmacy, Qiqihar Medical University, Qiqihar 161006 China; b School of Pharmacy, Qiqihar Medical University, Qiqihar 161006 China; c The Institute of Astragalus Industry Research, Qiqihar Medical University, Qiqihar 161006 China

**Keywords:** *Astragalus membranaceus* flower, Mixing schemes, Ions’ quantity, Ultrasonic-assisted extraction, Flavonoids

## Abstract

Traditional extraction under merely one specific solvent was confronted with incomplete phytochemical unscramble problem. In view of this, in order to obtain the overall chemical understanding, we attempted to use *Astragalus membranaceus* flower, an abundant exploitable resource, to screen a novel extraction mixing scheme via gradient solvents based on the ions’ quantity detected in UHPLC-Q-TOF-MS/MS approach. Samples were firstly extracted by different concentration ethanol, and then, six mixing schemes were investigated and one scheme with maximum detected ions was screened out. After identification procedures based on an comprehensive reference database, 136 components covered 54 flavonoids were accurately identified, indicating that flavonoids may played a critical role in *Astragalus membranaceus* flower. Through the Box–Behnken Design optimization, 29.79 mg/g of flavonoids were extracted under ethanol concentration of 35 %, solid–liquid ratio of 1:50, extraction time of 50 min. The experiments showed that the established mixing scheme could obtain more comprehensive ingredients compared to orthodox extraction, further guide the optimization of significative ingredients scientifically. The present paper could promote the development of *Astragalus membranaceus* flower.

## 1 Introduction

*Astragalus membranaceus*, one kind of precious rhizomatic medicine mainly distributed in North of China, exhibited multifarious functions like immunity enhancement, anti-hyperglycemic, anti-inflammatory, anti-cancer, anti-oxidant and anti-viral [1]. Due to its important role, the demand of *Astragalus membranaceus* was radically increasing [2], wild *Astragalus membranaceus* could not meet the needs. Hence, artificial *Astragalus membranaceus* cultivations include *Astragalus membranaceus* (Fisch.) Bge. var. *mongholicus* (Bge.) P. K. Hsiao and *Astragalus membranaceus* (Fisch.) Bug were widely planted, and the former one catched more attention for its large output and favourable quality. During the growting of *Astragalus membranaceus* plant, plenty of flowers were picked off in order to reduce the nutrients’ consumption of root, resulting in a great waste of plant resources. In some areas of China, *Astragalus membranaceus* flower was deemed as one kind of delicious food or a tonic tea, nevertheless, the consumption amount was especially little. Therefore, broaden the exploitation scopes of *Astragalus membranaceus* flower appeared to be extremely urgent. In order to make the plentiful application of *Astragalus membranaceus* flower, utilizing *Astragalus membranaceus* flower into suitable functional materials was meaningful more than ever. As for further bioactive functional study, phytochemical unscramble was the first priority of *Astragalus membranaceus* flower. Up to now, the chemical study of *Astragalus membranaceus* flower was abnormal scarce, few extent study showed that there were some flavonoids [3], however, the overall chemical profiles were not reported yet.

**Fig. 1:**
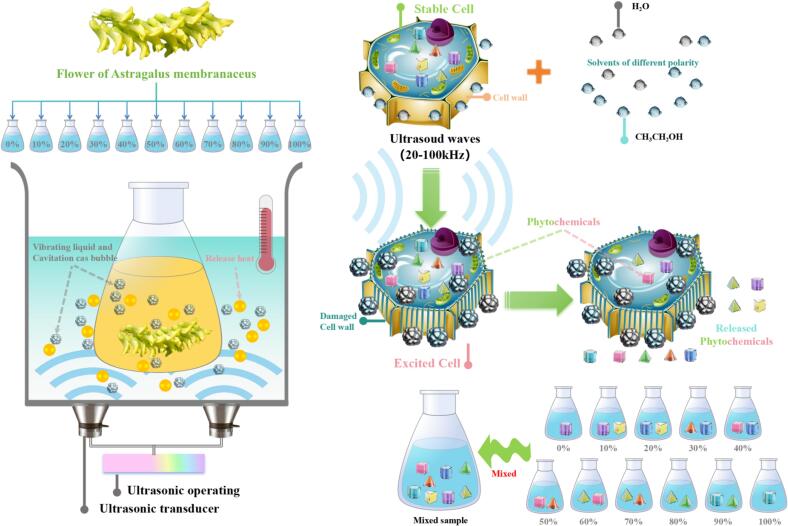
The mechanism of action in *Astragalus membranaceus* flower.

As for phytochemical understanding, extraction method played an important role in the preliminary acquisition. Ultrasound-assisted extraction, a developed extraction method with low cost and high efficiency, was widely used for extracting the abundant constituents from plant and its by-product [4], [5]. During ultrasound-assisted process, the interspace of plant cell increased or even rupture under high-speed cavitation and stirring effect, making the components better contacting between solid and the liquid [6]. When mentioned the constituents’ extraction, there maybe some constituents linked with heat-labile protein in plant, causing the extraction difficulty under traditional methods. Therefore, under powerful supersonic wave with indoor temperature, some connecting key between small compounds and protein maybe unlocked, so that obtained more phytochemical ingredients [7]. Besides, as for chemical extraction, the dissolution was directly correlated to the polarity of solvent [8]. Usually, ethanol with low-polarity and water with high-polarity were the recommended solvents in the extraction procedure. Nevertheless, merely one specific-polarity solvent could not gained the all-sided components. Hence, solvent with different proportion, which showed stepwise polarity, maybe helpful for the dissolving more constituents. In order to roundly elucidate the chemical profiles of *Astragalus membranaceus* flower, the fire-new mixture means based on gradient ethanol extractions may showed more approving extraction effect for multitudinous constituents [2]. The action mechanism was displayed in Fig. 1.

For the past few years, UHPLC-Q-TOF-MS/MS approach has been a favourable technique to reveal the chemical components in multiple scopes [9], [10]. Recently, with the rapid development of visible data software and reference database, the applications of UHPLC-Q-TOF-MS/MS has been extensived in the unscramble of multifarious ingredients *in vitro* and *in vivo* [11], [12]. Therefore, high sensitive UHPLC-Q-TOF-MS/MS tool combined with appropriate sample preparation could be a novel means to comprehensively uncover the chemical profiles of *Astragalus membranaceus* flower. In this paper, in order to explore the phytochemistry profiles of *Astragalus membranaceus* flower more comprehensively, UHPLC-Q-TOF-MS/MS was used to characterize the chemical components. And then, the nominated flavones constituents with large proportion based on the identification results were further extracted by an innovative gradient solvent with different polarity via an ultrasound-assisted equipment. The present data could provid the infarctate evidence for the exploitation of *Astragalus membranaceus* flower. Importantly, this paper also offered a superior extraction means for roundly uncovering the meaningful ingredients of *Astragalus membranaceus* flower and other Chinese herbs.

## 2 Experimental methods

### 2.1 Materials and reagents

*Astragalus membranaceus* flower collected from the medicinal garden in Qiqihar Medical University was identified as the flower of *Astragalus membranaceus* (Fisch.) Bge. var. *mongholicus* (Bge.) P. K. Hsiao by Doctor Qi Liu. Methanol, acetonitrile and formic acid were of HPLC-grade. Methanol, acetonitrile were obtained from Merck company (Darmstadt, Germany), and formic acid was offered by Thermo Fisher Scientific (Pittsburgh, PA, USA). Experimental distilled water was purchased from A.s. Watson Group.. Rutin reference was of HPLC-grade and the purity was greater than 98 %. Other reagents including ethanol, sodium nitrite, aluminium nitrate, sodium hydroxide were of analytical grade.

### 2.2 Characterization of constituents from *Astragalus membranaceus* flower

#### 2.2.1 Preliminary sample preparation

*Astragalus membranaceus* flower was dried at 50 ℃ in a drying oven, and then crushed by a micro-plant grinder (Tianjin Test Instrument Co., Ltd.) and pass through 50 mesh. Totally 11 of 0.5 g *Astragalus membranaceus* flower was extracted by an ultrasound-assisted equipment (power 200 W, frequency 30 kHz) in 30 mL of different gradient of ethanol (water, 10 % ethanol, 20 % ethanol, 30 % ethanol, 40 % ethanol, 50 % ethanol, 60 % ethanol, 70 % ethanol, 80 % ethanol, 90 % ethanol, absolute ethanol) for 50 min, respectively. Finally, 11 samples covering constituents of gradient polarity were obtained.

#### 2.2.2 Mixing schemes of samples

All the above-mentioned 11 samples were mixed in different ways to screen an appropriate scheme with full-scale constituents of different polarity. Next, six kinds of common mixed schemes based on isopyknic solution were obtained by different orders. Then, the befitting amount of the mixed solution was taken out and filtered throgh a 0.22 µm membrane. Finally, the six samples from different schemes were severally detected by an UHPLC-Q-TOF-MS/MS system.

#### 2.2.3 Optimization of UHPLC-Q-TOF-MS/MS conditions

In this study, a rapid ultra-performance liquid chromatography system (LC-30A, Shimadzu Corporation in Kyoto, Japan) coupled with a high-sensitive mass spectrometer (Triple TOF 5600, AB Sciex Corporation in Redwood City, California, USA) was applied for data acquiration. In order to optimize the liquid chromatography and mass spectrometry process, the parameters were selected as follows.

Conditions for liquid chromatography: an ACQUITY^TM^ UPLC HSS T_3_ ultra-high performance separation column (Waters Corporation, 100 mm × 2.1 mm id, 1.8 µm) was used for the separation of multiple ingredients. The column’s temperature was maintained at 40℃. Distilled water(A) and acetonitrile(B), both consisting 1 ‰ of formic acid, were choosed as the mobile phases for liquid separation. Gradient elution procedures were setting as below: 0.01–3 min, 5 %-15 % B; 3–3.5 min, 15 %-15 % B, 3.5–6 min, 15 %-30 % B; 6–6.5 min, 30 %-30 % B; 6.5–9 min, 30 %-50 % B; 9–10 min, 50 %-50 % B; 10–15 min, 50 %-75 % B; 15–20 min, 75 %-100 % B. The flow rate of mobile phase was maintained at 0.4 mL/min and the sample inject volume was of 5 µL.

Parameters of mass spectrum: electrospray ionization was adopted in mass spectrum system, and the temperature was maintained at 500℃ during the positive ion mode(POS) and negative ion mode(NEG) data collection courses. Both under POS and NEG acquiration, voltages of ionspray voltage floating were set at 5500 V and −4500 V, severally. When acquire the TOFMS information, the mass range was between 50 Da to 1500 Da under 80 ms accumulation. The gas pressures of nebulizer, auxiliary and curtain were held at 55psi, 55psi and 35psi, respectively. And the voltages of declustering potential(DP) and collision energy (CE) were kept at 100 V and 10 V. Concurrently, MS/MS data was obtained through the information dependent acquisition, and the voltage of CE was controlled at 20 V-60 V. Under the aforesaid optimized parameters, both MS and MS/MS data were obtained synchronously for further data processing.

#### 2.2.4 Identification of phytochemical profiles

The obtained data files were leaded in MSDIAL4.92 for the extraction, matching and alignmen of peaks. Then a matrix including comprehensive information such as rentention time, *m*/*z* and area of peaks were obtained after removing the excrescent isotope data. Based on the ions’s quantity, the appropriate data was screened and matched with the standard database (all in one database) for non-target research. Finally, the identification results were confirmed based on satisfying the corresponding ions in database less than 5mDa and MS/MS matching score greater than 70 simultaneously.

### 2.3 Extraction optimizing of flavonoids in *Astragalus membranaceus* flower

#### 2.3.1 Establishment of standard curve

Totally 10.46 mg of rutin was accurately weighed and dissolved in 50 mL of 60 % ethanol, then a reference solution with 0.21 mg/mL was obtained. Next, different volumes (1 mL, 2 mL, 3 mL, 4 mL, 5 mL, 6 mL, 7 mL and 8 mL) of reference solution were taken out and placed in 25 mL volumeter bottles, 1 mL of 5 % sodium nitrite liquid was added. After shaking and placing for 6 min, 1 mL of 10 % aluminium nitrate was added and placing for 6 min. Finally, 10 mL of 4 % sodium hydroxide liquid was added, and then filled with disstilled water to the scale mark. After reacting for 10 min, the absorption value was determined at 510 nm by an UV–Vis spectrophotometer (Shanghai Aosi Scientific Instrument Co., Ltd.). The standard curve was drawn with mass concentration of rutin (X, mg/mL) as the horizontal coordinate and light absorption value Y as the vertical coordinate.

#### 2.3.2 Single factor experimental design

Ethanol concentration (10 %, 20 %, 30 %, 40 %, 50 %, 60 %, 70 %, 80 %, 90 %), solid–liquid ratio (1:20, 1:30, 1:40, 1:50, 1:60) and extraction time (10 min, 20 min, 30 min, 40 min, 50 min, 60 min) were orderly screened to analyze the effects of these factors on the contents of total flavonoids by an ultrasonic equipment. Each experiment was repeated three times.

#### 2.3.3 Response surface experiment

Ethanol concentration(X1), solid–liquid ratio(X2) and extraction time(X3) were selected as the independent variables. The effects on the extraction rate of total flavonoids were investigated. 17 factorial tests and 5 central point replicates were conducted. Total flavonoid content(TFC) was selected as the response to combination of arguments. The follow quadratic polynomial equation was applied during the multiple regression analysis. In the equation, Y was the predicted response value, β_0_ was a constant coefficient. Besides, β_i_, β_ii_ and β_ij_ were the linear coefficients, quadratic terms, and interaction coefficients, respectively. X_i_ and X_j_ were the encoded values of independent variables (i ≠ j). K represented the counts of independent variables.

$$Y{=\beta }_{0}+\sum_{i=1}^{k}\beta_{i}X_{i}+\sum_{i=1}^{k}\beta_{\mathrm{ii}}X_{i}^{2}+\sum_{i=1}^{k-1}\sum_{j=i+1}^{k}\beta_{\mathrm{ij}}X_{i}X_{j}$$

## 3 Results and discussion

### 3.1 Comprehensive scheme for ingredients undersatnding in *Astragalus membranaceus* flower

**Fig. 2:**
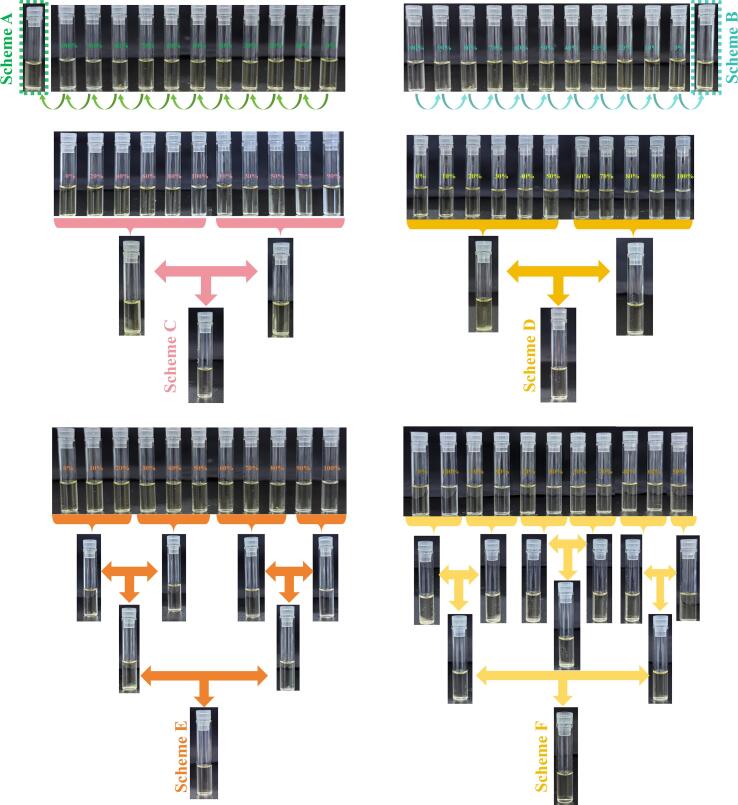
Different mixing schemes among the extracted samples under different gradient ethanol of *Astragalus membranaceus* flower.

Ultrasonic-assisted, one of the important extraction method in plant, was widely applied attributed to its high extraction ratio and nice convenience in recent years [13]. In order to extracted the components in *Astragalus membranaceus* flower efficiently, ultrasonic-assisted equipment was used for the extraction of *Astragalus membranaceus* flower. In addition, solvent was especially important for the extraction [14]. Due to the multiformity of ingredients, simple one solvent could not extracted them absolutely. As for diffeent compounds, the solubility were different. However, the dissolution of the compounds follows the principle of “similar phase dissolution”, once the polarity of compound and solvent were similar, the dissolution will be with high level. For extraction, the familiar solvents in analyzing scope were water, methanol and ethanol [15]. Among them, water showed well extraction of high-polarity substances with inexpensive and non-poisonous advantages, however, the extraction efficiency of low-polarity substances was very low. Methanol exhibited preferable extraction of low-polarity substances, but it may resulted in high toxicity and cost. As for ethanol, it not only owned ideal extraction of low-polarity substances, but also showed low cost and non-toxicity. From the existing references we found that, plant was conventionaly extracted by one specific solvent [16]. However, that may resulted in the ingredients extracted incomprehensively, for one solvent could not dissolve all constituents with different polarities. Thus it could be seen that one full-view scheme based on different polarity solvents was particularly important for roundly chemical understanding. In view of this, we established an innovatory extraction method as follows. Different gradient extraction solvents including water (0 % ethanol), 10 % ethanol, 20 % ethanol, 30 % ethanol, 40 % ethanol, 50 % ethanol, 60 % ethanol, 70 % ethanol, 80 % ethanol, 90 % ethanol and 100 % ethanol were apartly used for extraction. Traditionally, in order to obtain the full-scale components with different polarity, every sample should be detected and identified laboriously. Fortunately, the employed UHPLC-Q-TOF-MS/MS approach owned high-sensitivity advantage, helping the ions responsed well under extremely low concentration. Hence, a mixing sample composed all the several solutions was a feasible manner. Nevertheless, during the mixing process, some constituents maybe dissoved out [17], resulting the ions detected decreased. Therefore, in order to unscramble the maximum components, 6 kinds of mixing schemes among the above-mentioned eleven samples were conducted for ion’s screening analysis. The detail mixing procedures were displayed in Fig. 2.

**Fig. 3:**
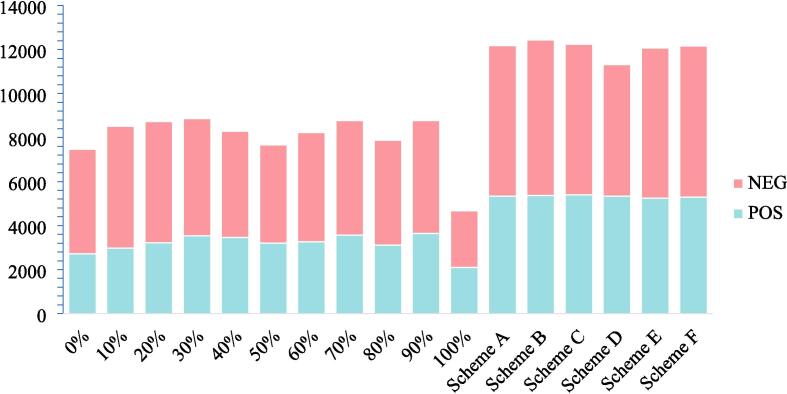
Quantities of detected ions in different samples based on UHPLC-Q-TOF-MS/MS tool.

When refered to the identification, the amount of identified compunds was closely related to the quantity of nominated detected ions [18]. The greater the number of MS ions, the more the identification results based on MS/MS information. Hence, the contrastive analysis of ions’ quantities summation under positive ion mode(POS) and negative ion mode(NEG) of samples from eleven single solvent and six mixing schemes were carried out under appropriate threshold value. The results were displayed in Fig. 3. As shown in Fig. 3, compared to samples from single solvent, samples from six mixing schemes were significantly increased, indicating that mixing strategy has a great advantage for more identification results. Besides, as for the six mixing schemes, scheme B could obtained the largest ions, indicating that scheme B was more forceful for further all-sided identification. Therefore, sample from scheme B was selected for further identification.

### 3.2 Rapid identification of compounds in *Astragalus membranaceus* flower extraction

**Fig. 4:**
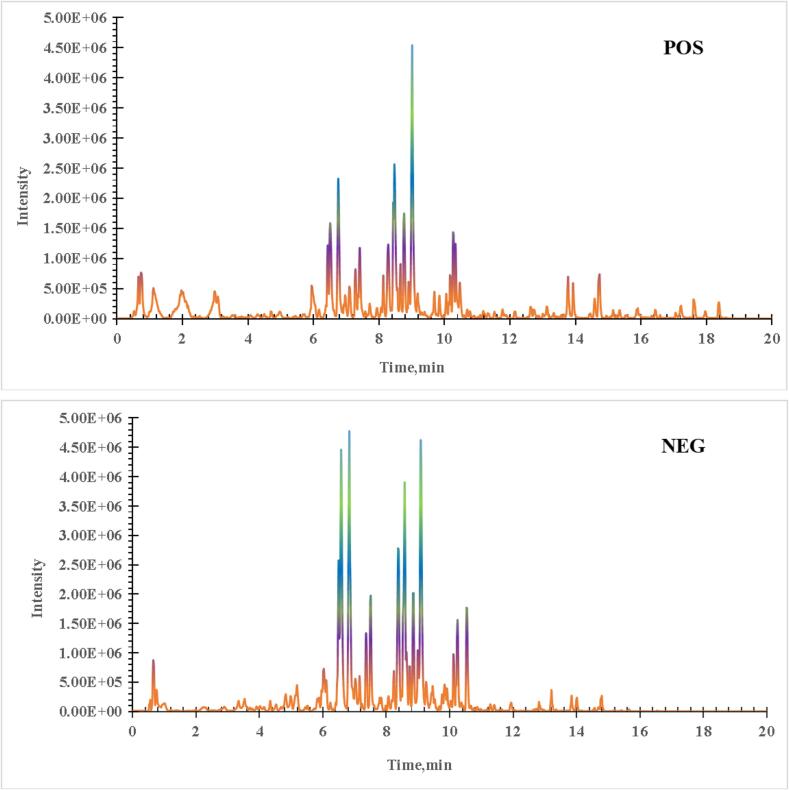
The base peak ion chromatograms of *Astragalus membranaceus* flower based on UHPLC-Q-TOF-MS/MS approach.

The preprocessed sample from scheme B covered the full-scale chemical ingredients was analyzed by UHPLC-Q-TOF-MS/MS and the approving base peak ion chromatograms(BPCs) were obtained in Fig. 4. As displayed in Fig. 4, the detected peaks were with good sheap peaks, high response and good separation, indicating that the optimized conditions of liquid phase and mass spectrum were satisfactory.

**Fig. 5:**
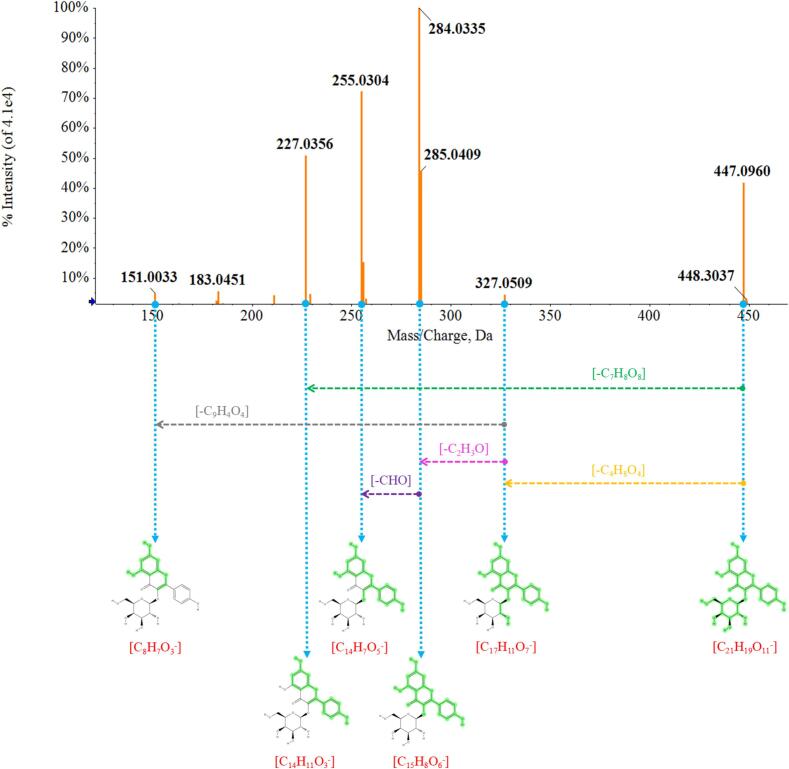
The detailed fragmentation process of kaempferol 3-ss-D-galactoside under negative ion mode by UHPLC-Q-TOF-MS/MS.

Based on the credible BPCs, the original data under POS and NEG was analyzed by PeakView and MarkerView and then confirmed by MS/MS database library. Take the identification process of kaempferol 3-ss-D-galactoside for example. The retention time was 6.955 min in NEG mode, the excimer ion peak was 447.0960 Da, the molecular formula was calculated as C_21_H_20_O_11_ in terms of the elemental composition rule, indicating the current residue maybe C_21_H_19_O_11_-. Through the careful observation of MS/MS fragments, C_21_H_19_O_11_- may lose -C_4_H_8_O_4_ and then formed the residue C_17_H_11_O_7_- (*m*/*z* 327.0509), furher lose –C_2_H_3_O and obtained the residue C_15_H_8_O_6_- (*m*/*z* 284.0335), then lose –CHO and gained C_14_H_7_O_5_- (*m*/*z* 255.0304). Besides, C_17_H_11_O_7_- (*m*/*z* 327.0509) maybe directly lose -C_9_H_4_O_4_ and became C_8_H_7_O_3_- (*m*/*z* 151.0033). In addition, there was still a possibility that C_21_H_19_O_11_- lose C_7_H_8_O_8_- and formed the residue C_14_H_11_O_3_- (*m*/*z* 227.0356). Based on these above information, the ion was preliminarily estimated as kaempferol 3-ss-D-galactoside. Then, after matching with the reference database, the ion was finally confired as kaempferol 3-ss-D-galactoside doubtlessly. The detailed fragmentation process was shown in Fig. 5. According to this identification process, 136 of nominated ions were finally identified successfully. The detail information of identified ingredients including retention time, *m*/*z*, CAS number, compound’s name, molecular formula and detail classify under POS and NEG was shown in Table 1.

**Table 1:** The identified polar chemical profiles of *Astragalus membranaceus* flower based on UHPLC-Q-TOF-MS/MS approach.

No.	Rt (min)	*m*/*z*	CAS number	Name	Adduct type	Formula	Mass Error (mDa）	Class
1	0.56	177.0987	543–38-4	L-canavanine	[M + H]^+^	C_5_H_12_N_4_O_3_	0.50	Amino acids
2	0.59	154.0643	71–00-1	L-histidine	[M − H]^−^	C_6_H_9_N_3_O_2_	1.42	Amino acids
3	0.62	175.1189	74–79-3	L-arginine	[M + H]^+^	C_6_H_14_N_4_O_2_	−0.02	Amino acids
4	0.62	113.0383	70–47-3	L-asparagine	[M − H_2_O − H]^−^	C_4_H_8_N_2_O_3_	2.17	Amino acids
5	0.62	104.0367	56–45-1	L-serine	[M − H]^−^	C_3_H_7_NO_3_	0.74	Amino acids
6	0.67	138.0555	535–83-1	trigonelline	[M + H]^+^	C_7_H_7_NO_2_	0.51	Alkaloids
7	0.67	135.0326	3909–12-4	threonic acid	[M − H]^−^	C_4_H_8_O_5_	2.32	Sugar acids
8	0.70	136.0624	73–24-5	adenine	[M + H]^+^	C_5_H_5_N_5_	0.64	Purines
9	0.73	130.0866	1723–00-8	D-pipecolinic acid	[M + H]^+^	C_6_H_11_NO_2_	0.64	Amino acids
10	0.74	133.0173	6915–15-7	DL-malic acid	[M − H]^−^	C_4_H_6_O_5_	2.77	Hydroxy acids
11	0.75	307.0806	3371–27-5	(−)-gallocatechin	[M + H]^+^	C_15_H_14_O_7_	−1.13	Flavonoids
12	0.78	103.0549	122–78-1	phenylacetaldehyde	[M + H − H_2_O]^+^	C_8_H_8_O	1.18	Phenylacetaldehydes
13	0.81	182.0797	60–18-4	L-tyrosine	[M + H]^+^	C_9_H_11_NO_3_	−1.74	Amino acids
14	0.84	132.1022	73–32-5	L-isoleucine	[M + H]^+^	C_6_H_13_NO_2_	0.13	Amino acids
15	0.98	191.0225	77–92-9	citric acid	[M − H]^−^	C_6_H_8_O_7_	2.16	Amino acids
16	1.07	243.0647	58–96-8	uridine	[M−H]-	C_9_H_12_N_2_O_6_	2.07	Pyrimidine nucleosides
17	1.08	123.0442	123–08-0	4-hydroxybenzaldehyde	[M + H]^+^	C_7_H_6_O_2_	0.19	Aldehydes
18	1.08	136.0758	104–14-3	DL-octopamine	[M + H − H_2_O]^+^	C_8_H_11_NO_2_	0.28	Phenols
19	1.16	117.0219	516–05-2	methylmalonic acid	[M − H]^−^	C_4_H_6_O_4_	2.18	Amino acids
20	1.57	153.0396	69–89-6	xanthine	[M + H]^+^	C_5_H_4_N_4_O_2_	−0.93	Imidazopyrimidines
21	1.88	137.0275	99–06-9	3-hydroxybenzoic acid	[M − H]^−^	C_7_H_6_O_3_	2.37	Benzoic acids
22	1.93 /1.94	149.0592 /131.0490	140–10-3	*trans*-cinnamic acid	[M + H]^+^/ [M + H − H_2_O]^+^	C_9_H_8_O_2_	−0.29/ 0.07	Cinnamic acids
23	1.94	166.0863	150–30-1	DL-phenylalanine	[M + H]^+^	C_9_H_11_NO_2_	−0.33	Amino acids
24	2.55	231.1707	41017–96-3	ile-val	[M + H]^+^	C_11_H_22_N_2_O_3_	0.58	Amino acids
25	2.57	163.0392	331–39-5	caffeic acid	[M + H − H_2_O]^+^	C_9_H_8_O_4_	0.22	Hydroxycinnamic acids
26	2.87	127.0389	67–47-0	5-(hydroxymethyl)-2-furaldehyde	[M + H]^+^	C_6_H_6_O_3_	−0.01	Aldehydes
27	2.98	205.0973	73–22-3	L-tryptophan	[M + H]^+^	C_11_H_12_N_2_O_2_	−0.39	Indoles
28	2.98	146.0600	1074–86-8	1H-indole-4-carboxaldehyde	[M + H]^+^	C_9_H_7_NO	0.18	Indoles
29	3.26	261.1441	2566–39-4	N-L-γ-glutamyl-L-leucine	[M + H]^+^	C_11_H_20_N_2_O_5_	−0.46	Amino acids
30	3.34/ 3.43	147.0443/163.0432	501–98-4	p-coumaric acid	[M + H − H_2_O]^+^/ [M − H]^−^	C_9_H_8_O_3_	−0.03/ 2.9	Hydroxycinnamic acids
31	3.56	190.0489	492–27-3	kynurenic acid	[M + H]^+^	C_10_H_7_NO_3_	−0.49	Quinolines
32	3.60	627.1547	29125–80-2	quercetin 3,4′-diglucoside	[M + H]^+^	C_27_H_30_O_17_	−1.41	Flavonoids
33	3.61	139.0387	99–96-7	4-hydroxybenzoic acid	[M + H]^+^	C_7_H_6_O_3_	0.54	Benzoic acids
34	3.64	305.0679	13425–13-3	epigallocatechin	[M − H]^−^	C_15_H_14_O_7_	1.13	Flavonoids
35	3.73	175.0627	3237–44-3	2-isopropylmalic acid	[M − H]^−^	C_7_H_12_O_5_	1.13	Fatty Acyls
36	3.76	195.0540	13382–27-9	galactonic acid	[M − H]^−^	C_6_H_12_O_7_	2.77	Hydroxy acids
37	3.79	295.1297	20556–22-3	L-Phenylalanine, L-α-glutamyl-	[M + H]^+^	C_14_H_18_N_2_O_5_	0.03	Amino acids
38	3.85	177.0543	537–98-4	*trans*-ferulic acid	[M + H − H_2_O]^+^	C_10_H_10_O_4_	−0.32	Hydroxycinnamic acids
39	4.06	325.0967	2446–60-8	*cis*-melilotoside	[M − H]^−^	C_15_H_18_O_8_	3.57	Glycosyl compounds
40	4.07	163.0432	614–60-8	*trans*-2-hydroxycinnamic acid	[M − H]^−^	C_9_H_8_O_3_	2.55	Hydroxycinnamic acids
41	4.11	265.1547	3918–91-0	valylphenylalanine	[M + H]^+^	C_14_H_20_N_2_O_3_	0.33	Amino acids
42	4.28	465.1017	482–36-0	hyperin	[M + H]^+^	C_21_H_20_O_12_	−1.84	Flavonoids
43	4.28	627.1566	99224–12-1	herbacetin-3,8-diglucopyranoside	[M + H]^+^	C_27_H_30_O_17_	0.86	Flavonoids
44	4.30	159.0698	111–16-0	pimelic acid	[M − H]^−^	C_7_H_12_O_4_	2.96	Fatty Acyls
45	4.42	177.0214	486–35-1	7,8-dihydroxycoumarin	[M − H]^−^	C_9_H_6_O_4_	1.69	Hydroxycinnamic acids
46	4.43	433.1331	325144–72-7	licoagroside B	[M + H]^+^	C_18_H_24_O_12_	−1.31	Saccharolipids
47	4.86	611.1604	52187–80-1	luteolin-7,3′-di-O-glucoside	[M + H]^+^	C_27_H_30_O_16_	−0.98	Flavonoids
48	4.88	119.0496	10597–60-1	(3,4-dihydroxyphenyl)ethanol	[M + H − 2H_2_O]^+^	C_8_H_10_O_3_	0.80	Phenols
49	5.16	172.1013	54896–21-8	N-acetyl-D-norleucine	[M − H]^−^	C_8_H_15_NO_3_	2.73	Amino acids
50	5.40	137.0589	31982–85-1	(3aS,7aS)-hexahydro-2-benzofuran-1,3-dione	[M + H − H_2_O]+	C_8_H_10_O_3_	−0.81	Isobenzofurans
51	5.43	481.0978	652–78-8	gossypin	[M + H]^+^	C_21_H_20_O_13_	−0.12	Flavonoids
52	5.45	121.0281	69–72-7	salicylic acid	[M + H − H_2_O]^+^	C_7_H_6_O_3_	−0.01	Benzoic acids
53	5.62	135.0477	99–93-4	4′-hydroxyacetophenone	[M − H]^−^	C_8_H_8_O_2_	2.21	Ketones
54	5.87	479.0855	15648–86-9	myricetin 3-galactopyranoside	[M − H]^−^	C_21_H_20_O_13_	1.28	Flavonoids
55	5.89	319.0449	529–44-2	myricetin	[M + H]^+^	C_15_H_10_O_8_	−0.49	Flavonoids
56	5.95	597.1450	142905–18-8	quercetin-3-O-β-D-xylopyranosyl (1 → 6)-β-D-glucopyranoside	[M + H]^+^	C_26_H_28_O_16_	−0.67	Flavonoids
57	5.96	303.0493	117–39-5	quercetin	[M + H]^+^	C_15_H_10_O_7_	−1.22	Flavonoids
58	5.97	317.0656	520–11-6	6-methoxyluteolin	[M + H]^+^	C_16_H_12_O_7_	−0.19	Flavonoids
59	5.97	663.1523	259234–17-8	rhamnetin 3-sophoroside	[M + Na]^+^	C_28_H_32_O_17_	−1.04	Flavonoids
60	6.02	611.1600	153–18-4	rutin	[M + H]^+^	C_27_H_30_O_16_	−1.22	Flavonoids
61	6.04	173.0860	505–48-6	suberic acid	[M − H]^−^	C_8_H_14_O_4_	3.58	Fatty Acyls
62	6.17/ 6.18	208.0957/206.0835	2018–61-3	N-acetyl-L-phenylalanine	[M + H]^+^/[M − H]^−^	C_11_H_13_NO_3_	−1.33/ 0.95	Amino acids
63	6.22	206.0863	10172–89-1	N-acetyl-D-phenylalanine	[M − H]^−^	C_11_H_13_NO_3_	3.73	Amino acids
64	6.32/ 6.31	491.1209/447.1284	20633–67-4	calycosin-7-O-glucoside	[M + H]^+^/ [M + FA − H]−	C_22_H_22_O_10_	0.03/ 0.64	Flavonoids
65	6.38	595.1658	28447–29-2	kaempferol 3-rungioside	[M + H]^+^	C_27_H_30_O_15_	−0.06	Flavonoids
66	6.43	245.0966	87–32-1	N-acetyltryptophan	[M − H]^−^	C_13_H_14_N_2_O_3_	3.06	Amino acids
67	6.44	579.1379	110352–79-9	kaempferol 3-O-vicianoside	[M − H]^−^	C_26_H_28_O_15_	0.86	Flavonoids
68	6.50	465.1035	20229–56-5	spiraeoside	[M + H]^+^	C_21_H_20_O_12_	−0.04	Flavonoids
69	6.75	617.1468	16310–92-2	datiscin	[M + Na]^+^	C_27_H_30_O_15_	−1.4	Flavonoids
70	6.82	121.0331	65–85-0	benzoic acid	[M − H]^−^	C_7_H_6_O_2_	2.81	Benzoic acids
71	6.96	447.0963	23627–87-4	kaempferol 3-ss-D-galactoside	[M − H]^−^	C_21_H_20_O_11_	2.26	Flavonoids
72	6.96	449.1083	480–10-4	astragalin	[M + H]^+^	C_21_H_20_O_11_	0.46	Flavonoids
73	6.96	287.0559	480–15-9	datiscetin	[M + H]^+^	C_15_H_10_O_6_	0.40	Flavonoids
74	7.07	477.1062	1085711–35-8	5,7-dihydroxy-2-(4-hydroxy-3-methoxyphenyl)-4-oxo-4H-chromen-3-yl hexopyranoside	[M − H]^−^	C_22_H_22_O_12_	1.16	Flavonoids
75	7.07	479.1194	5041–82-7	isorhamnetin 3-O-glucoside	[M + H]^+^	C_22_H_22_O_12_	4.39	Flavonoids
76	7.13	533.1284	137705–39-6	6′'-O-malonylglycitin	[M + H]^+^	C_25_H_24_O_13_	−1.4	Isoflavonoids
77	7.23	463.1243	17680–84-1	hispiduloside	[M + H]^+^	C_22_H_22_O_11_	−0.12	Flavonoids
78	7.23	625.1762	116183–66-5	complanatoside	[M + H]^+^	C_28_H_32_O_16_	−0.12	Flavonoids
79	7.24	187.0992	123–99-9	azelaic acid	[M − H]^−^	C_9_H_16_O_4_	1.23	Fatty Acyls
80	7.33	165.0590	828–01-3	DL-3-phenyllactic acid	[M − H]^−^	C_9_H_10_O_3_	2.78	Phenylpropanoic acids
81	7.40	317.0652	480–19-3	isorhamnetin	[M + H]^+^	C_16_H_12_O_7_	0.03	Flavonoids
82	7.75 /7.74	475.1270/431.1335	486–62-4	ononin	[M + H]^+^/ [M + FA − H]^−^	C_22_H_22_O_9_	−1.56/ 1.58	Isoflavonoids
83	8.10	447.1295	40246–10-4	glycitin	[M + H]^+^	C_22_H_22_O_10_	1.56	Isoflavonoids
84	8.11	609.1813	55653–37-7	rhamnocitrin 3-rutinoside	[M + H]^+^	C_28_H_32_O_15_	−0.18	Flavonoids
85	8.16	255.0654	486–66-8	daidzein	[M + H]^+^	C_15_H_10_O_4_	−0.25	Isoflavonoids
86	8.26	477.1082	569–90-4	nepetin 7-glucoside	[M − H]^−^	C_22_H_22_O_12_	3.61	Flavonoids
87	8.35	247.1319	21293–29-8	(+)-abscisic acid	[M + H − H_2_O]^+^	C_15_H_20_O_4_	−1.36	Prenol lipids
88	8.40	301.0708	2284–31-3	pratensein	[M + H]^+^	C_16_H_12_O_6_	−0.52	Isoflavonoids
89	8.60	299.0219	6468–55-9	demethylwedelolactone	[M − H]^−^	C_15_H_8_O_7_	2.10	Isoflavonoids
90	8.75/8.76	463.1252/461.1130	611–40-5	tectoridin	[M + H]^+^/[M − H]^−^	C_22_H_22_O_11_	1.25/ 3.05	Isoflavonoids
91	8.76	301.0719	80453–44-7	padmatin	[M + H − H_2_O]^+^	C_16_H_14_O_7_	0.73	Flavonoids
92	8.77	577.1605	1137424–36-2	5-hydroxy-2-(4-hydroxyphenyl)-7-methoxy-4-oxo-4H-chromen-3-yl 2-O-(6-deoxy-α-L-mannopyranosyl)-α-L-arabinofuranoside	[M − H]^−^	C_27_H_30_O_14_	2.38	Flavonoids
93	8.84	493.1343	25474–11-7	jaceoside	[M + H]^+^	C_23_H_24_O_12_	−1.4	Flavonoids
94	8.99	301.0714	20243–59-8	hydroxygenkwanin	[M + H]^+^	C_16_H_12_O_6_	0.40	Flavonoids
95	9.16	471.3459	76964–07-3	esculentic acid	[M + H − H_2_O]^+^	C_30_H_48_O_5_	−1.40	Prenol lipids
96	9.21	236.1268	17966–67-5	N-benzoyl-DL-leucine	[M + H]^+^	C_13_H_17_NO_3_	−0.28	Amino acids
97	9.24	273.0758	480–41-1	naringenin	[M + H]^+^	C_15_H_12_O_5_	0.89	Flavonoids
98	9.31	271.0602	446–72-0	genistein	[M + H]^+^	C_15_H_10_O_5_	−0.25	Isoflavonoids
99	9.36	215.1312	1852–04-6	undecanedioic acid	[M − H]^−^	C_11_H_20_O_4_	2.29	Fatty Acyls
100	9.38	327.2212	51146–90-8	(10E,15Z)-9,12,13-trihydroxyoctadeca-10,15-dienoic acid	[M − H]^−^	C_18_H_32_O_5_	3.36	Fatty Acyls
101	9.66	329.0687	22697–65-0	4′,5,7-trihydroxy-3,6-dimethoxyflavone	[M − H]^−^	C_17_H_14_O_7_	2.20	Flavonoids
102	9.67	227.1316	6402–36-4	*trans*-traumatic acid	[M − H]^−^	C_12_H_20_O_4_	2.58	Fatty Acyls
103	9.75	331.0817	33429–83-3	3,4′-dimethoxy-5,7,3′-trihydroxyflavone	[M + H]^+^	C_17_H_14_O_7_	0.00	Flavonoids
104	9.77	469.3315	18449–41-7	madecassic acid	[M + H − 2H_2_O]^+^	C_30_H_48_O_6_	0.18	Prenol lipids
105	9.77	329.2369	1217866–47-1	(9Z)-5,8,11-trihydroxyoctadec-9-enoic acid	[M − H]^−^	C_18_H_34_O_5_	3.48	Fatty Acyls
106	9.91	257.0810	69097–97-8	liquiritigenin	[M + H]^+^	C_15_H_12_O_4_	−0.31	Flavonoids
107	9.92	255.0690	961–29-5	2′,4,4′-Trihydroxychalcone	[M − H]^−^	C_15_H_12_O_4_	2.52	Chalcones
108	9.95	327.2207	2111873–96-0	(12Z,15Z)-7,9,10-trihydroxyoctadeca-12,15-dienoic acid	[M − H]^−^	C_18_H_32_O_5_	2.93	Fatty Acyls
109	10.04	229.1467	693–23-2	dodecanedioic acid	[M − H]^−^	C_12_H_22_O_4_	1.64	Fatty Acyls
110	10.04	235.2058	57499–57-7	1-[1,6-dimethyl-4-(4-methyl-3-pentenyl)-3-cyclohexen-1-yl]ethanone	[M + H]^+^	C_16_H_26_O	−0.06	Prenol lipids
111	10.11	267.0692	140439–35-6	6-hydroxy-3′-methoxyflavone	[M − H]^−^	C_16_H_12_O_4_	2.71	Flavonoids
112	10.11	269.0809	485–72-3	formononetin	[M + H]^+^	C_16_H_12_O_4_	−0.70	Isoflavonoids
113	10.23/10.23	315.0546/317.0667	90–19-7	rhamnetin	[M − H]^−^/[M + H]^+^	C_16_H_12_O_7_	3.36/ 0.33	Flavonoids
114	10.30	301.1064	73340–41-7	3-hydroxy-9,10-dimethoxypterocarpan	[M + H]^+^	C_17_H_16_O_5_	−0.58	Isoflavonoids
115	10.31	230.2474	1643–20-5	N,N-dimethyldodecylamine N-oxide	[M + H]^+^	C_14_H_31_NO	−0.68	Aminoxides
116	10.42	303.1214	64474–51-7	isomucronulatol	[M + H]^+^	C_17_H_18_O_5_	−2.07	Isoflavonoids
117	11.18	285.0764	491–80-5	biochanin A	[M + H]^+^	C_16_H_12_O_5_	0.40	Isoflavonoids
118	11.32	301.0720	520–34-3	diosmetin	[M + H]^+^	C_16_H_12_O_6_	2.13	Flavonoids
119	11.40	313.0748	53948–00-8	odoratin	[M − H]^−^	C_17_H_14_O_6_	2.84	Isoflavonoids
120	11.42	283.0649	20575–57-9	calycosin	[M − H]^−^	C_16_H_12_O_5_	3.48	Isoflavonoids
121	11.57	293.2105	67204–66-4	chromomoric acid B	[M + H]^+^	C_18_H_28_O_3_	−0.73	Fatty Acyls
122	11.82	277.2167	87984–82-5	13S-hydroxy-9Z,11E,15Z-octadecatrienoic acid	[M + H − H_2_O]^+^	C_18_H_30_O_3_	0.00	Fatty Acyls
123	13.63	285.2107	505–54-4	hexadecanedioic acid	[M − H]^−^	C_16_H_30_O_4_	3.51	Fatty Acyls
124	13.72	353.2696	18465–99-1	1-monolinolenin	[M + H]^+^	C_21_H_36_O_4_	−0.03	Fatty Acyls
125	14.32	313.2740	542–44-9	1-palmitoylglycerol	[M + H − H_2_O]^+^	C_19_H_38_O_4_	−0.24	Glycerolipids
126	14.71	279.2321	138231–04-6	laetisaric acid	[M + H − H_2_O]^+^	C_18_H_32_O_3_	−0.24	Fatty Acyls
127	15.14	277.2174	54739–30-9	13-keto-9Z,11E-octadecadienoic acid	[M + H − H_2_O]^+^	C_18_H_30_O_3_	0.55	Fatty Acyls
128	15.88	279.1596	1962–75-0	1,4-dibutyl benzene-1,4-dicarboxylate	[M + H]^+^	C_16_H_22_O_4_	0.16	Benzoic acids
129	15.88	149.0236	85–44-9	phthalic anhydride	[M + H]^+^	C_8_H_4_O_3_	0.11	Benzofurans
130	16.43	324.2910	68171–52-8	linoleoyl ethanolamide	[M + H]^+^	C_20_H_37_NO_2_	0.61	Amines
131	17.48	271.2320	764–67-0	2-hydroxypalmitic acid	[M − H]^−^	C_16_H_32_O_3_	4.18	Fatty Acyls
132	17.62	279.2327	16833–54-8	pinolenic acid	[M + H]^+^	C_18_H_30_O_2_	0.43	Fatty Acyls
133	18.38	282.2802	301–02-0	oleamide	[M + H]^+^	C_18_H_35_NO	0.73	Fatty Acyls
134	18.60	281.2474	506–21-8	linoelaidic acid	[M + H]^+^	C_18_H_32_O_2_	−0.21	Fatty Acyls
135	18.80	357.3001	111–03-5	monoolein	[M + H]^+^	C_21_H_40_O_4_	−0.49	Glycerolipids
136	19.30	593.2771	15664–29-6	pheophorbide A	[M + H]^+^	C_35_H_36_N_4_O_5_	−0.67	Tetrapyrroles

**Fig. 6:**
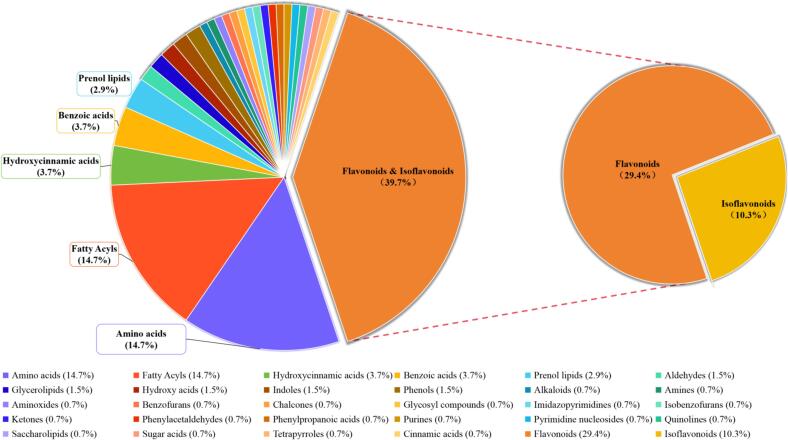
The detailed proportions of the identified compounds with different classification.

On account of the close relationship between constituents and bioactivity, chemical’s in-depth interpretation was particularly important for further funtional study [19]. Hence, the detailed classification of the identified ingredients were conducted and displayed in Fig. 6. As Fig. 6 showed, up to 30 kinds of components were screened out in *Astragalus membranaceus* flower, covering flavonoids, isolavonoids, amino acids, fatty acyls, *etc.*, meaning that the chemical profiles was diversiform in *Astragalus membranaceus* flower. As hundreds of different chemical ingredients in *Astragalus membranaceus* flower, the functional material basis maybe related to a few kinds of chemical profiles. Among the identified ingredients, the quantity’s proportion of flavonoids(covered isoflavonoids) occupyed up to 39.7 %, which was the highest level. illustrating that flavonoids may played a critical role in the bioactive understanding of *Astragalus membranaceus* flower. As was well known, flavonoids has attracted wide attention in different scopes due to their affluent bioactivities [18], [19]. In *Astragalus membranaceus* flower, the identified flavonoids such as hyperin, myricetin, rutin, pratensein, ononin, glycitin, *etc.* were also reported manifold physiologically-actives. For example, hyperin could suppresses renal inflammation through regulating macrophage polarization in mice with type 2 diabetes mellitus [20], exhibited well effect on non-alcoholic fatty liver disease through cholesterol metabolism and bile acid metabolism [21], imposed a blocking or reversing effect on hepatic fibrosis via againsting inflammation [22]. Myricetin could reduce the production of inflammatory cytokine, and then give play to ameliorating pancreatitis [23]. Rutin could inhibited the advanced glycation end products-stimulated inflammatory reaction [24]. Pratensein could alleviate the oxidative stress and NLRP3 inflammasome activation in OGD/R-injured HT22 cells [25]. Ononin could ameliorates inflammation and cartilage degradation in rat chondrocytes with IL-1β-induced osteoarthritiss [26]. Glycitin was reported antioxidant activity in pentylenetetrazol induced spasticity animal model [27]. These recent researches showed that the identified flavonoids from *Astragalus membranaceus* flower exhibited obvious anti-inflammatory and antioxidant activities, guiding the direction for further functional study. Therefore, the flavonoids’ extraction was an important step for the deepgoing development of *Astragalus membranaceus* flower. In order to pertinently extract the flavonoids in *Astragalus membranaceus* flower, an efficient ultrasonic-assisted method combined with single factor and Box-Behnken Design tests was conducted to optimize the appropriate extracting conditions.

### 3.3 Extraction optimizing of flavonoids in *Astragalus membranaceus* flower

#### 3.3.1 Investigation of single factor

**Fig. 7:**
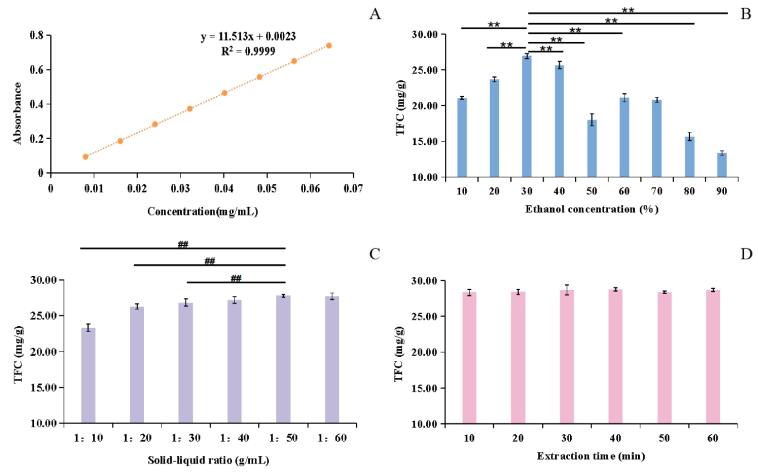
The effect of single factor on the extraction of total flavonoids from *Astragalus membranaceus* flower. Note: A: The linear relation between concentration and absorbance of rutin; B: Effect of ethanol’s concentration on TFC; C: Effect of solid–liquid ratio on TFC; D: Effect of Extraction time on TFC. **: p < 0.01, highly significant difference, TFC of 30 % ethanol vs others. ##: p < 0.01, highly significant difference, TFC of 1:50 vs others.

UV–Vis absorption was widely used for the the evaluation of content [28]. Based on color reaction, the linear results of rutin and investigation results of single factors were displayed in Fig. 7. From Fig. 7-A, the equation of linear regression was as follows: Y = 11.513X + 0.0023 (R^2^ = 0.9999), meaning that the linear relationship was applicable in the concentration range of 0.01 ∼ 0.07 μg/mL, indicating that the data of samples will be available durng this period. Under the estiblished method, different parameters of ethanol concentration, solid–liquid ratio and extracting time were conducted in the extraction process and the effect of these factors on the yield of flavonoids were as well as shown in Fig. 7-B, Fig. 7-C and Fig. 7-D.

As the identified ingredients shown, the identified flavonoid covered its glycosides and aglycones. In general, compared to flavonoid aglycones, flavonoid glycosides exhibited higher polarity [29]. Besides, the longer the linked carbohydrate chain, the greater the polarity was [30]. As for flavonoid aglycones, organic slovents such as ethanol, methanol, ethyl acetate, *etc.* could exhibit favourable solubility. However, flavonoid aglycones were hardly dissolved in the distilled water. As for flavonoid glycosides, solvents covered water, ethanol, methanol, *etc.* could dissolved out well. In view of the solvent’s safety and cost, different concentration of ethanol, which could give consideration to both flavonoid glycosides and flavonoid aglycones [31], was selected as the solvent for the comprehensive extraction of flavonoids in *Astragalus membranaceus* flower. As could be seen from Fig. 7-B, under the conditions of fixed solid–liquid ratio of 1:30 (g:mL) and extraction time of 30 min, the dissolution of total flavonoids gradually increased and then reached the maximum value at 30 % of ethanol. When refered to the different concentration of ethanol for flavonoids’ extraction, the dissolution rates of flavonoid glycosides and flavonoid aglycones were with different proportions in every scetion of extracting solvent. The result indicated that the dissolution’s proportion of flavonoid glycosides and flavonoid aglycones was compatible under 30 % ethanol, which contributed the highest level of flavonoids. Hence, 30 % of ethanol was selected as the solvent for further extraction screening.

The effect of solid–liquid ratio on the extraction of total flavonoids was shown in Fig. 7-C. Under the fixed conditions of 30 % ethanol and extraction time of 30 min, the extraction amount of total flavonoids gradually increased with the increase of solid–liquid ratio. However, when the solid–liquid ratio was 1:50, the TPC was in high level and changed slightly subsequently. Therefore, the solid–liquid ratio of the extraction of total flavonoids was finally selected at 1:50 under 30 % ethanol.

The active substances may be favorablely dissolved under applicable extraction time. The effects of extraction time on the extraction rates of TFC were shown in Fig. 7-D. With other extraction parameters (30 % ethanol, solid–liquid ratio 1:50) unaltered, different extraction times were carried out. The TFC gradually increased with the increase of extraction time, and the content was the highest under 40 min. This showed that 40 min was the befitting extraction time under 30 % ethanol.

#### 3.3.2 Box-Behnken Design predicted model and statistical analysis

On the basis of the preliminary single factor experiment’s results, the three independent variables including ethanol concentration, solid–liquid ratio and extraction time were selected. The response surface method was used to furtherly optimize the three variables for 17 factorial tests, the experimental data was showed in Table 2. Based on Design-Expert 12.0 software, multiple linear regression and binomial fitting equations were respectively:

Y1 = 7.55 + 0.41*A + 0.88*B-0.45*C + 0.0048*A*B + 0.0019*A*C-0.0013*B*C-0.01*A^2^-0.0096*B^2^ + 0.0059*C^2^

**Table 2:** The total flavonoid contents of *Astragalus membranaceus* flower by ultrasonic-assisted extraction using Box Behnken Design.

No.	Extraction conditions						TFC (mg·g^−1^)
	Code variables			Decoded variables			
	X1	X2	X3	Ethanol concentration(%)	Solid-liquid ratio (g:mL)	Extraction time(min)	
1	−1	−1	0	20	1:40	40	26.03
2	1	−1	0	40	1:40	40	26.54
3	−1	1	0	20	1:60	40	25.60
4	1	1	0	40	1:60	40	28.05
5	−1	0	−1	20	1:50	30	26.78
6	1	0	−1	40	1:50	30	28.99
7	−1	0	1	20	1:50	50	26.85
8	1	0	1	40	1:50	50	29.84
9	0	−1	−1	30	1:40	30	28.15
10	0	1	−1	30	1:60	30	28.24
11	0	−1	1	30	1:40	50	28.43
12	0	1	1	30	1:60	50	27.99
13	0	0	0	30	1:50	40	28.45
14	0	0	0	30	1:50	40	28.93
15	0	0	0	30	1:50	40	28.96
16	0	0	0	30	1:50	40	28.37
17	0	0	0	30	1:50	40	28.13

X1: Ethanol concentration (%), X2: solid–liquid ratio (g:mL), X3: extraction time (min).

F, P and R2 values were applied for the evaluation of the quadratic model, the predicted responses of TFC and relevant independent variables of second-order polynomial equations were analyzed by variance analysis. The F-values of Y1 were calculated as 10.13, and the P-values were less than 0.05, indicating that the model was statistically significant (Table 3). According to F value, the effects of factors on Y1 was as follows: ethanol concentration > solid–liquid ratio > extraction time. On the basis of P value, ethanol concentration has a significant effect on Y1[31]. The model determination coefficients was 0.9287, explaining that the correlation was benifit, the experimental data and predicted data were approximate. The F value of the missing fit for Y1was 2.49, illustrating that the missing fit to pure error was not significant. Summing up the above, these results showed that the accuracy of the polynomial model was sufficient. Finally, the TPC was determined under the screened extraction conditions in order to verify the predicted model.

**Table 3:** Analysis of variance for the regression equation.

Sources	F-Value	p-Value
Model	10.13	0.003
A-A	38.46	0.0004
B-B	0.31	0.5963
C-C	0.52	0.4945
AB	4.36	0.0751
AC	0.72	0.4244
BC	0.33	0.5814
A^2^	21.53	0.0024
B^2^	18.10	0.0038
C^2^	6.94	0.0337
Lack of Fit	2.49	0.1993
R^2^	0.9287	

Note: The P value less than 0.05 was significant.

#### 3.3.3 Response surface analysis

**Fig. 8:**
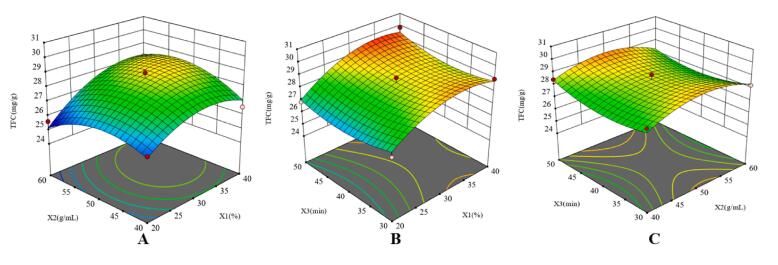
Optimization of TFCs of *Astragalus membranaceus* flower. Note: (A): The interaction between ethanol concentration and liquid-to-solid ratio; (B): The interaction between ethanol concentration and extraction time; (C): The interaction between liquid-to-solid ratio and ultrasonic. (X1): Ethanol concentration, (X2): Liquid-to-solid ratio, (X3): Extraction time.

The visual three-dimensional response surface diagrams of pairwise interaction analysis among ethanol concentration(X1), solid–liquid ratio(X2) and ultrasonic time(X3) on the interactions of TFC from *Astragalus membranaceus* flower were conducted and displayed in Fig. 8. Under one fixed factor, the variation of TFC between other factors were drawed. Fig. 8-A showed the interactive effect between ethanol concentration and solid–liquid ratio under the fixed ultrasonic time, TFC was significantly increased and then reduced, the contour was circular, indicating that the interaction of ethanol concentration and solid–liquid ratio on the extraction rate of TFC was not significant. The response surfaces in Fig. 8(B) and Fig. 8(C) were relatively smooth, meaning that the influences of ethanol concentration and ultrasonic time, solid–liquid ratio and ultrasonic time on the extraction rate of TFC were not obvious, which was as well as consistent with the results in the ANOVA result. Finally, based on the Box-Behnken Design, the optimal conditions were determined as follows: ethanol concentration of 36.09 %, solid–liquid ratio of 1:51.31, extraction time of 50.0 min. The predicted extraction of total flavonoids was 29.65 mg/g under these parameters.

#### 3.3.4 Optimization and model verification

In order to ensure the feasibility of actual operation, the extraction conditions were modified as the approximate integer values: ethanol concentration of 35 %, solid–liquid ratio of 1:50, extraction time of 50.0 min. Through the further confirmatory experiments(n = 3), the TFC were 29.79 mg/g under the affirmative parameters, the results were displayed in Table 4. The content of actual extraction was closed to the theoretical value, and the RSD value of three repeating verification experiments was 0.88 %, which indicated that the model was well fitted and the parameters were highly reliable. Summing up the above, TFC could be preferable through 50 times of 35 % ethanol by extracting 50 min. Therefore, the optimized technology process could be an applicable extraction course for the flavonoids ingredients in *Astragalus membranaceus* flower.

**Table 4:** The total flavonoid content of *Astragalus membranaceus* flower under the optimized technology(n = 3).

Experiment number	TFC(mg/g)	Mean(mg/g)	RSD(%)
1	29.78	29.79	0.89
2	29.53		
3	30.06		

## 4 Conclusion

Chemical composition of medical plant was complex and varied, different constituents may exhibit distinct polarity due to diverse molecular structure. Hence, appropriate extraction solvent was particularly important for ingredients’ dissolution rate. However, sample used for chemical unscramble was usually simply extracted by one fixed-polarity solvent, resulting in the one-sided identification results on account of the imperfect dissolution. In this work, we developed an mixing scheme covered overal alternative ions based on the gradient extracting solvents with different polarities via effecient ultrasonic-assisted extraction. The suggested innovative strategy could be a novel method for the identification of full-scale polar components in plant, especially with infrequent references in existing research scope. Through the preliminary extraction of gradient ethanol, ingredients with inequable polarities were dissolved out as comprehensive as possible. Compared to single extraction, mixing procedure could dramatically improve the quantity of detected ions via an high throughput UHPLC-Q-TOF-MS/MS. The most founding was that scheme B detecting the maximum ions was confirmed as the sample preparation method for further identification in consideration of interferential precipitating during the mixing operation. The chemical identification and the classification showed that flavonoids which owned obvious anti-inflammatory activity may played an positive role in further functional utilization of *Astragalus membranaceus* flower. Based on this, ultrasound-assisted extraction was used for the elaborate optimization of potential active flavonoids from *Astragalus membranaceus* flower by single factor experiment and Box-Behnken design. After extracting for 50 min with 50 times of 35 % ethanol, the extraction of total flavonoids was up to 29.79 mg/g. The optimized extraction technology was high-efficiency and low-cost, which provided significant data for further purification and biological exploration. Summing up the above, the established method in this paper offered a fire-new means for comprehensive phytochemical understanding and efficient extraction of target components in *Astragalus membranaceus* flower, guided further development especially in potential anti-inflammatory and antioxidant fields.

## CRediT authorship contribution statement

**Yumei Wang:** Writing – review & editing, Writing – original draft, Supervision, Methodology, Funding acquisition, Formal analysis, Data curation. **Meiling Gu:** Writing – original draft, Visualization, Software, Resources, Investigation, Formal analysis. **Meng Zhang:** Validation, Supervision, Resources, Investigation, Data curation. **Jialin Mao:** Validation, Resources, Investigation. **Yujian Han:** Supervision, Resources, Investigation. **Qi Liu:** Writing – review & editing, Writing – original draft, Visualization, Validation, Supervision, Software, Resources, Project administration, Methodology, Investigation, Funding acquisition, Formal analysis, Data curation, Conceptualization.

## Declaration of competing interest

The authors declare that they have no known competing financial interests or personal relationships that could have appeared to influence the work reported in this paper.
